# Genome-Wide Identification and Expression Analysis of the LbDof Transcription Factor Family Genes in *Lycium barbarum*

**DOI:** 10.3390/plants14111567

**Published:** 2025-05-22

**Authors:** Yuchang Wang, Hongrui Wang, Weinan Li, Guoli Dai, Jinhuan Chen

**Affiliations:** 1 College of Biological Sciences and Technology, Beijing Forestry University, Beijing 100083, China; y.c.wang420@outlook.com (Y.W.); k107234568@163.com (H.W.); liweinan1996@outlook.com (W.L.); 2 State Key Laboratory of Efficient Production of Forest Resources, Beijing Forestry University, Beijing 100083, China; 3 National Wolfberry Engineering Research Center, Ningxia Academy of Agriculture and Forestry Sciences, Yinchuan 750002, China

**Keywords:** *Lycium barbarum*, Dof transcription factors, abiotic stress, fruit development, genome identification

## Abstract

*Lycium barbarum*, a nutrient-rich fruit known for its resilience to drought and high salinity, presents an opportunity to explore stress tolerance at the molecular level. This study explores the molecular mechanisms underlying stress tolerance and fruit development in *L. barbarum* by characterizing its Dof transcription factor family. Through genomic analysis, 39 *LbDof* genes were identified, with their structural, phylogenetic, and physicochemical properties systematically examined. *Cis*-acting regulatory element analysis revealed motifs associated with growth, stress, light, and hormone responses, while expression profiling demonstrated organ-specific patterns and significant upregulation under drought and saline–alkaline stress. Additionally, dynamic expression changes were observed across fruit development stages, suggesting regulatory roles in maturation. Phylogenetic classification grouped *LbDof* genes into ten subgroups, with chromosomal mapping indicating segmental duplications as a key evolutionary driver. Furthermore, the study offers a comprehensive genomic and functional analysis of *LbDof* genes, highlighting their potential roles in stress adaptation and fruit maturation. The findings provide a theoretical basis for breeding stress-resistant crops and insights into enhancing plant resilience.

## 1. Introduction

*Lycium barbarum* (wolfberry; 2n = 2x = 24) is a nutrient-rich fruit renowned for its abundance of bioactive compounds, including polysaccharides, betaine, and flavonoids [1]. Used for centuries in traditional Chinese medicine, it is known for its ability to nourish yin, strengthen the kidneys, enhance vision, and delay aging [2]. This perennial shrub, native to northwestern China [3], exhibits remarkable adaptability to extreme environmental conditions such as drought and high salinity. Its resilience and economic value make it a promising candidate for cultivation in saline–alkaline soils of arid and semi-arid regions. However, the underlying molecular mechanisms governing its stress tolerance and fruit development remain largely unexplored.

Transcription factors (TFs) play a pivotal role in regulating gene expression in response to environmental stresses, thereby controlling plant growth, development, metabolic responses, and adaptability. Among these, Dof (DNA-binding with One Finger) is a unique family of plant-specific transcription factors that are crucial in various physiological and developmental processes [4]. Dof TFs are specialized proteins found in plants. They contain a conserved C2/C2 zinc-finger domain, a unique structure that allows them to bind to specific DNA sequences in plant gene promoter regions (5′-AAAG-3′) [5]. By attaching to these regions, Dof TFs regulate the expression of genes involved in stress responses, metabolic processes, and hormone signaling [6]. These proteins are essential for plant adaptation to abiotic stresses, including drought, salinity, and extreme temperatures. They achieve this through several mechanisms, including regulating water balance, scavenging harmful reactive oxygen species (ROS), and modulating hormone signaling pathways [7]. As a result, Dof TFs enhance plant growth and improve their ability to survive under stressful conditions.

With the continued advancement of plant genomics, systematic studies of the Dof TF family across various plant species have revealed its diverse roles in environmental adaptability [8,9]. Dof family members commonly arise from whole-genome and segmental duplications, with their number and distribution varying across plant species [10,11]. For instance, *Arabidopsis thaliana* contains 36 identified *Dof* genes [12], while *Zea mays*, *Camellia sinensis*, *Solanum lycopersicum*, and *Oryza sativa* contain 46, 29, 34, and 30 *Dof* genes, respectively [13,14,15,16]. The functions of these Dof TFs extend beyond regulating photoperiod responses, seed germination, and carbon–nitrogen metabolism to include the regulation of stress responses.

Dof TFs play diverse roles in plant responses to abiotic stresses, particularly drought and salinity. Research has demonstrated that GhDof1.7 in cotton (*Gossypium hirsutum*) enhances drought tolerance by regulating the expression of antioxidant genes [17]. This transcription factor increases the plant’s ability to scavenge ROS, thereby mitigating oxidative stress induced by water scarcity and preserving cellular function and structure [6]. In rice (*O. sativa*), OsDof1 promotes the synthesis of proline, which functions as an osmoprotectant to stabilize the internal environment of cells under water-deficit conditions [15]. In addition, multiple *Dof* genes in *Malus domestica* are significantly upregulated under drought stress, underscoring their key roles in water regulation and ion balance [18].

In response to salinity stress, Dof TFs also play critical roles. For example, GhDof1.7 activates defense mechanisms in cotton, facilitating ion transport and aiding the plant in adapting to salt stress [17]. In tomato, SlCDF3 enhances salt tolerance by regulating salt-responsive genes and optimizing ion homeostasis [19]. In Brassica rapa, Dof TFs regulate ABA-related genes, helping the plant manage salt stress by controlling water loss and modulating stomatal closure [20]. These studies demonstrate that Dof TFs enhance salt tolerance by regulating hormone signaling and maintaining ionic balance. Dof TFs not only regulate specific stress responses but also coordinate the interactions between multiple stress pathways. In *Arabidopsis*, CDF3 interacts with DREB and NAC TFs to regulate diverse stress-responsive genes and coordinate stress adaptation [12]. Dof TFs activate stress-responsive genes by binding to promoter elements such as the P-box motif. In cotton, GhDof1.7 targets P-box elements to upregulate antioxidant enzyme genes under drought and salt stress, mitigating oxidative damage [17].

Although Dof TFs have been extensively studied in many plant species, their specific roles in *L. barbarum* remain unclear. As a stress-tolerant plant capable of thriving in extreme environments, the stress response mechanisms of *L. barbarum* have not been fully elucidated. This study aims to systematically characterize the Dof TF family in *L. barbarum*, exploring their regulatory functions under natural growth conditions as well as under drought and saline–alkaline stress. Notably, this represents the first genome-wide identification and multi-omics integration of Dof TFs in *L. barbarum*, providing novel insights and candidate targets for the genetic improvement of stress-resilient crops. Through comparative genomics and transcriptomic analysis, we seek to reveal the functional characteristics of Dof TFs in *L. barbarum* and their roles in stress responses. A deeper understanding of these molecular mechanisms will provide a theoretical foundation for the genetic improvement of stress-tolerant crops like *L. barbarum* and offer valuable insights into developing strategies for enhancing plant resilience.

## 2. Results

### 2.1. Identification and Physicochemical Properties of LbDof

This study aimed to identify the Dof proteins encoded in the *L. barbarum* genome. A genome-wide search was conducted using BLASTp, with the query sequence being analyzed. The results from NCBI Batch CDD and Pfam were then confirmed using HMM profiles (PF02701). As a result, 39 LbDof proteins were identified (Table 1). These proteins were systematically named from LbDof 01 to LbDof 39 (Table 1). The lengths of the LbDof proteins ranged from 184 to 659 amino acids, with molecular weights varying from 19.88 kDa (LbDof 33) to 73.62 kDa (LbDof 09), averaging 35.80 kDa. Additionally, the isoelectric points (pI) exhibited significant variation, with LbDof 12 having the lowest pI (4.56) and LbDof 36 the highest (9.81). These findings suggest that the broad range of amino acid compositions, molecular weights, and isoelectric points of LbDof proteins may reflect their involvement in diverse biological processes.

### 2.2. Phylogenetic Analysis of LbDofs

**Figure 1:**
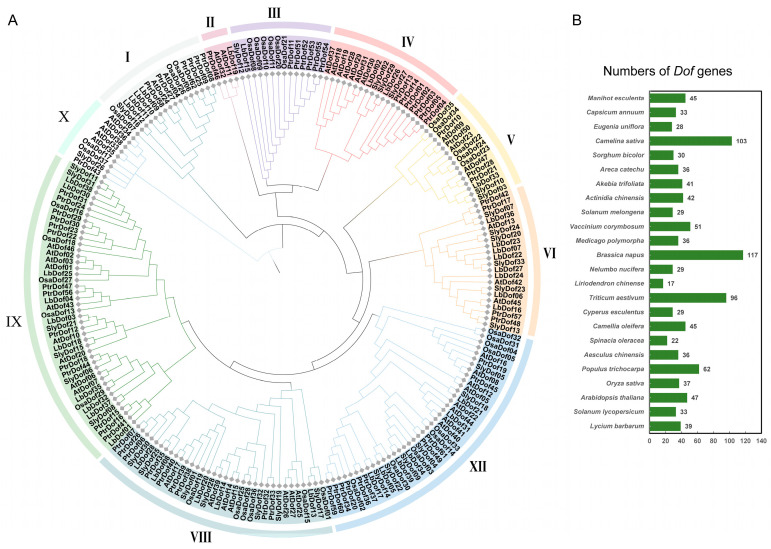
Phylogenetic analysis of the *Dof* gene family. (**A**) Phylogenetic reconstruction of *Dof* genes across *L. barbarum* (LbDof), *A. thaliana* (AtDof), *O. sativa* (OsaDof), *S. lycopersicum* (SlyDof), and *P. trichocarpa* (PtrDof), generated using MEGAX11 with 1000 bootstrap replicates. Branches are color-coded to delineate distinct clades, elucidating evolutionary divergence and relationships among Dof homologs; (**B**) number of genes for which phylogenetic analyses were constructed for each species.

To assess the evolutionary relationships among *Dof* genes, a neighbor-joining (NJ) phylogenetic tree was constructed using MEGA 11 software. The tree was generated from the Dof protein sequences of *L. barbarum* (39 LbDof), *S. lycopersicum* (33 SlyDof) *A. thaliana* (47 AtDof), *O. sativa* (37 OsaDof), and *P. trichocarpa* (62 PtrDof) (Figure 1B). The protein sequences of all Dofs used in the phylogenetic tree are shown in Table S1. The results revealed that the *Dof* genes exhibited significant diversity and were unevenly distributed across ten subgroups (I to X), with 16, 4, 14, 19, 15, 21, 39, 34, 44, and 9 members, respectively (Figure 1A). These findings highlight the utility of phylogenetic analyses in understanding the evolutionary relationships and functional potential of *Dof* genes. In conclusion, genomes with shared adaptive traits may exhibit similar functions, but further functional studies are required to confirm these relationships.

**Figure 2:**
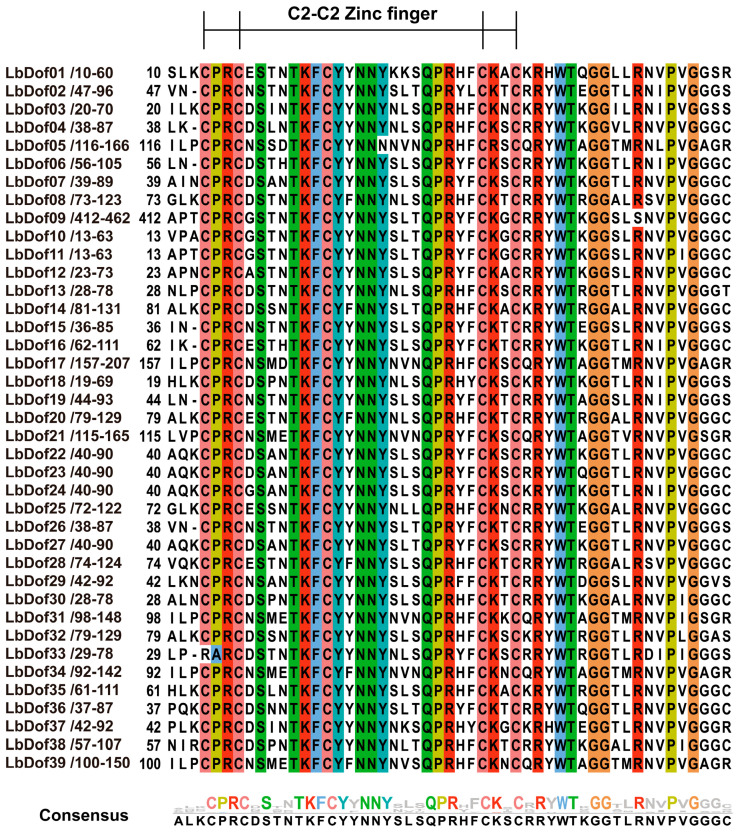
Conserved structural domains of LbDofs protein (colored regions indicate 100% amino acid homology); WebLogo shows conserved structural domains.

In addition, the amino acid sequences of the Dof structural domains in the 39 LbDof proteins were analyzed through multiple sequence comparisons. The results demonstrated that the structural domain sequences of LbDof proteins are highly conserved, with all sequences containing the typical C2/C2 motifs (Figure 2).

### 2.3. Chromosomal Location and Collinearity Analysis

**Figure 3:**
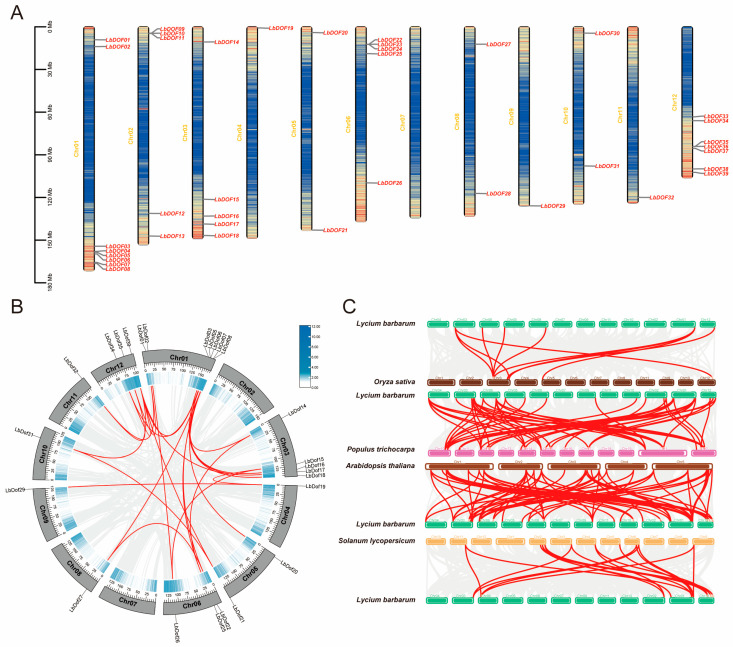
Chromosomal location and collinearity analysis. (**A**) Distribution and location of *LbDof* genes on 12 chromosomes of the wolfberry genome. The scale (Mb) indicates the length of the chromosome; (**B**) chromosomal location and gene duplication analysis of *LbDof* genes. The gray lines represent all co-lined blocks in the wolfberry genome; the red lines represent duplicated *LbDof* gene pairs. The heatmap in the outer circle represents the chromosomal gene density; (**C**) collinearity analysis between *Dof* genes in *L. barbarum* and four representative plants (*A. thaliana*, *O. sativa*, *P. trichocarpa*, and *S. lycopersicum*). Gray lines in the background represent blocks of covariance within the genome and the indicated plants and red lines highlight homologous *Dof* gene pairs.

Genome-wide identification revealed 39 *LbDof* genes in the *L. barbarum* genome, distributed across 11 chromosomes and named *LbDof01* to *LbDof39* based on their gene descriptions. Notably, no *LbDof* genes were identified on chromosome 7. A cluster of five *LbDof* genes (*LbDof03* to *LbDof08*) was found at the bottom of chromosome 1. Similarly, chromosome 6 contains a gene cluster spanning *LbDof22* to *LbDof25*, while chromosome 12 exhibits a higher density of *LbDof* genes, particularly near the bottom, where *LbDof33* to *LbDof39* are located. In contrast, chromosomes 4, 9, and 11 each contain only one *LbDof* gene (Figure 3A).

Gene duplication, including tandem and segmental duplication, is a key driver of gene family diversification and evolutionary adaptation. Our analysis identified 24 orthologous gene pairs among the 39 *LbDof* genes (Figure 3B). However, no tandem duplications were observed, indicating that segmental duplications are the primary mechanism driving the evolution of the *LbDof* gene family. Notably, only one duplicated gene pair was found on chromosome 9, and no duplicated pairs were detected on chromosomes 2 and 7 (Figure 3B).

A multicollinearity analysis compared the *L. barbarum* genome with those of *O. sativa*, *S. lycopersicum*, *P. trichocarpa*, and *A. thaliana*, (Figure 3C). This analysis revealed lineage-specific expansions during evolution. The highest collinearity was observed between *L. barbarum* and *A. thaliana* (69 orthologs), followed by *L. barbarum* and *P. trichocarpa* (60 orthologs) and *L. barbarum* and *S. lycopersicum* (13 orthologs), and the lowest was observed between *L. barbarum* and *O. sativa* (10 orthologs) (Figure 3C, Table S2). Among the chromosomes, *L. barbarum* chromosome 1 shared the most orthologs with the other species. Overall, the strong collinearity between *L. barbarum* and *A. thaliana* suggests that *Dof* genes are highly conserved and may share a common ancestor, except in cases of gene duplication or loss.

To assess selection pressure and divergence rates among the duplicated *LbDof* genes, we calculated the *Ka*/*Ks* ratios (Table S3). A *Ka*/*Ks* ratio > 1 indicates positive selection, *Ka*/*Ks* < 1 suggests purifying selection, and *Ka*/*Ks* = 1 implies neutral selection. Among the 24 homologous gene pairs identified in *L. barbarum*, all had *Ka*/*Ks* ratios below 1 (ranging from 0.11 to 0.46), indicating that these genes underwent purifying selection (Table S3). Additionally, the estimated divergence time for these duplicated genes ranged from 16.77 to 111.65 million years ago (Table S3).

### 2.4. Analyses of Gene Structures, Motifs, and Domains of LbDofs

**Figure 4:**
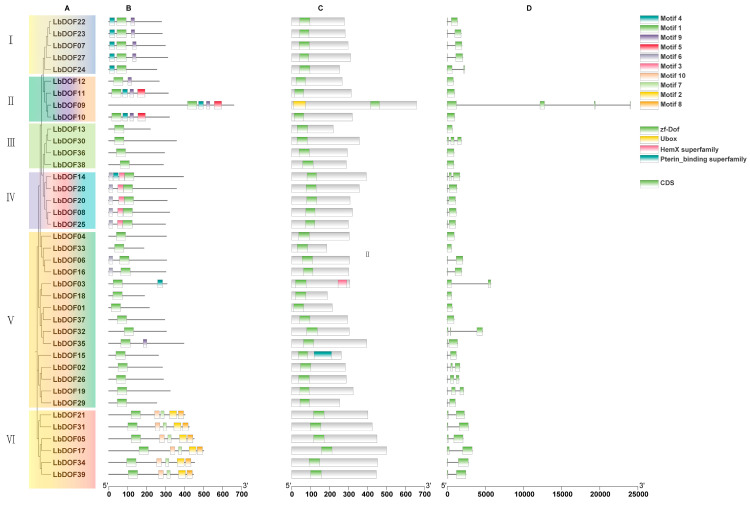
Analysis of phylogenetic relationships, gene structure, conserved structural domains, and conserved motifs of the *LbDof* gene. (**A**) Phylogenetic relationship analysis of the *LbDof* gene; (**B**) distribution of conserved protein motifs of Dofs; (**C**) predicted conserved protein domains of LbDofs; (**D**) exon–intron structures of *LbDofs*.

To investigate the relationships among the 39 *LbDof* genes, a phylogenetic tree was constructed using the neighbor-joining (NJ) method, classifying these genes into six subclades (I to VI) (Figure 4A). Subclade V was the largest, containing 15 *LbDof* genes, while subclades II and III were the smallest, each with only four members. Motif analysis revealed that all 39 *LbDof* genes contained between 1 and 5 conserved motifs, with motifs 1, 6, 9, and 10 being the most prominent. Notably, *LbDof21*, *LbDof31*, *LbDof05*, *LbDof17*, *LbDof34*, and *LbDof39* each contained a maximum of five motifs, while *LbDof11*, *LbDof09*, *LbDof10*, and *LbDof14* each contained four motifs (Figure 4B). These conserved motifs align with phylogenetic relationships, as members with similar motifs are clustered in the same clade, suggesting potential functional similarities. Conserved structural domain analysis confirmed that all *LbDof* genes contain the zf-Dof structural domain (Figure 4C). Gene structure analysis further showed that the number of introns in *LbDof* varied from 0 to 3 (Figure 4D). Genes within the same clade often exhibited similar intron-exon structures. For example, most genes in cluster I (5 genes) and VI (6 genes) had two exons (Figure 4D). Overall, the number of exons in each cluster ranged from one to four, with relatively simple coding regions (Figure 4D). *LbDof09* had the highest number of exons (four), while ten genes (*LbDof12*, *LbDof11*, *LbDof10*, *LbDof13*, *LbDof36*, *LbDof38*, *LbDof04*, *LbDof33*, *LbDof18*, *LbDof01*, and *LbDof37*) lacked introns (Figure 4D). These findings support the phylogenetic classification of the *LbDof* genes. In summary, the integration of phylogenetic analysis, cluster classification, conserved motif identification, and gene structure analysis indicates that LbDof proteins are highly conserved. Genes within the same clade may share similar functions, but further experimental studies are needed to validate these functional predictions.

### 2.5. Cis-Regulatory Element Analysis of LbDof Genes

**Figure 5:**
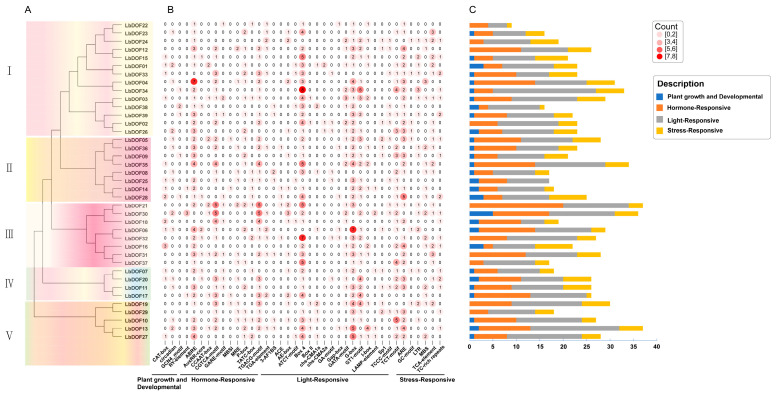
Schematic diagram of CAREs analysis of the *LbDof* gene promoter. (**A**) Promoter evolution tree of *LbDof* gene; (**B**) the shades of red and the numbers in the grid represent the number of corresponding CAREs; the darker the red color, the higher the number of CAREs; (**C**) bar graph showing the different types and numbers of the four CAREs of the *LbDof* gene promoter.

To further elucidate the transcriptional regulatory mechanisms, a *cis*-element analysis was conducted on the 2000 bp promoter region upstream of the *LbDof* gene coding sequences. The results (Table S4) identified a variety of CAREs, including those associated with growth and development, stress responses, light responses, and hormone responses. Notably, all *LbDof* gene promoters contained both light-responsive and hormone-responsive elements, suggesting that the majority of *Dof* genes may mediate life processes regulated by light or hormones. Furthermore, several MYB binding sites related to stress responses and flavonoid biosynthesis, such as MBS, MBSI, MRE, and CCAAT-box, were identified in the promoters of many *LbDof* genes. This indicates that these LbDof proteins may interact with MYB (Figure 5), participating in the regulation of drought stress responses, photoperiods, and flavonoid biosynthesis. Additionally, the majority of *LbDof* genes contained CAREs responsive to environmental stresses (ARE, GC-motif, TCA-element, TC-rich repeats, and LTR), suggesting their potential roles in stress responses such as low-temperature stress, drought stress, and wounding.

### 2.6. Subcellular Localization Analysis of LbDof

**Figure 6:**
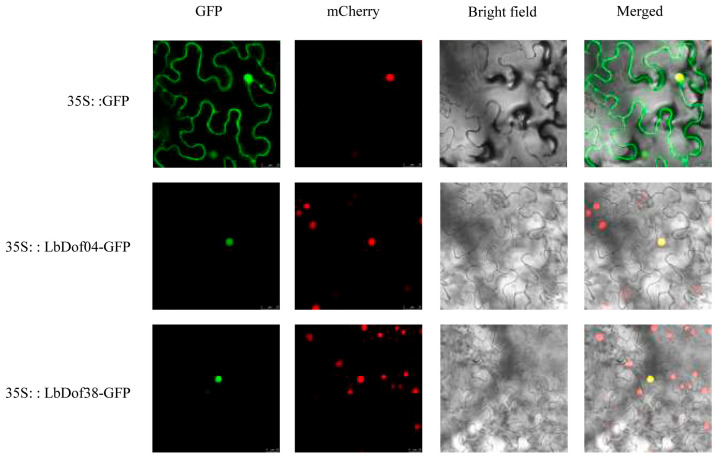
Subcellular localization of LbDof04 and LbDof38 in tobacco leaves. Based on the visualization of green fluorescent protein (GFP) in tobacco (*Nicotiana benthamiana*) leaves, LbDof04 and LbDof38 are localized in the nucleus. Bars = 25 μm.

According to the prediction of subcellular localization, all LbDof proteins were localized in the nucleus. To validate this prediction, we amplified the LbDof04 and LbDof38 genes and inserted them into the plant expression vector pbi121:35S. The results confirmed that both LbDof04 and LbDof38 proteins were localized in the nucleus, as the green fluorescent signals from LbDof04-eGFP and LbDof38-eGFP overlapped with the cytosolic autofluorescent signals and were concentrated in the nucleus (Figure 6).

### 2.7. Expression Pattern of LbDof in L. barbarum Under Different Organs

**Figure 7:**
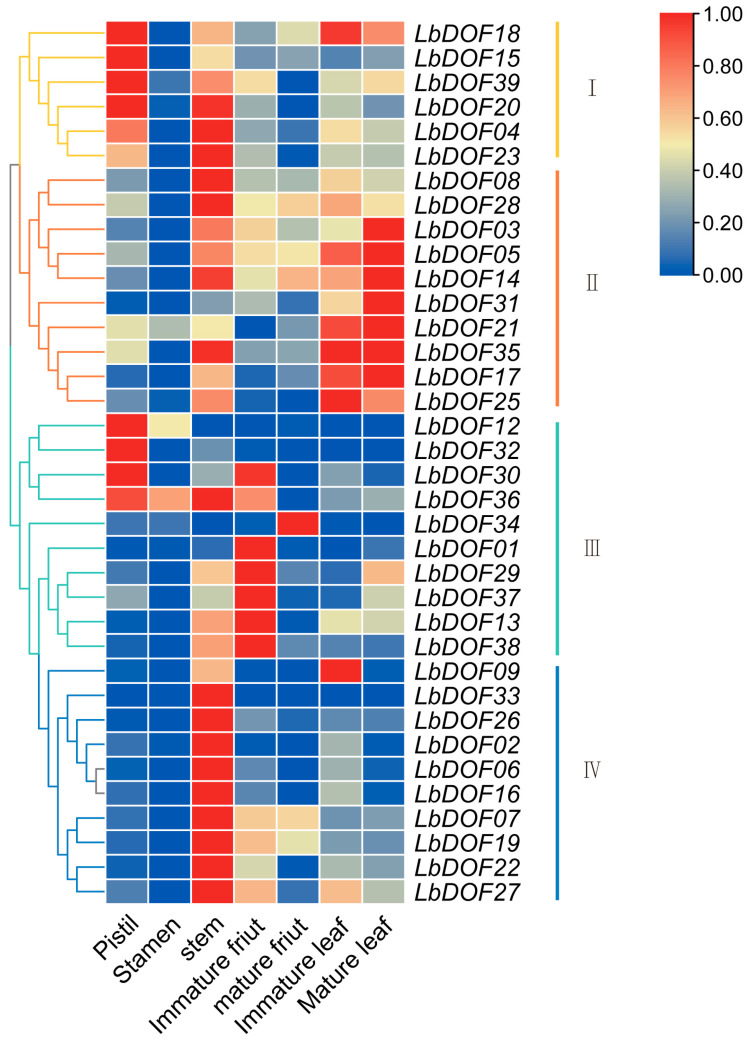
Heatmap of *LbDof* gene expression in different organs of *L. barbarum*. Relative expression levels are indicated by the color scale on the right side of the heat map. The colors in the legend represent log2 (FPKM + 1) values in different organs. The red color represents the maximum value and the blue color represents the minimum value.

To investigate the role of *LbDof* genes in the growth and development of *L. barbarum*, we analyzed the gene expression profiles across various organs, including pistils, stamens, stem segments, immature fruits, ripe fruits, immature leaves, and mature leaves, based on previous studies. The expression patterns of the *LbDof* gene family members revealed organ-specific variations. Among the 36 *Dof* genes analyzed, four distinct expression groups were identified. Notably, *LbDof18*, *LbDof15*, *LbDof39*, and *LbDof20* showed higher expression levels in the pistil. Except for *LbDof12* and *LbDof34*, the remaining 34 genes were expressed in the stem, with *LbDof33* being exclusively expressed in this organ. In contrast, *LbDof03*, *LbDof05*, *LbDof14*, *LbDof31*, *LbDof21*, *LbDof35*, and *LbDof17* were predominantly expressed in leaves. Additionally, *LbDof21*, *LbDof12*, *LbDof36*, and *LbDof34* were specifically expressed in stamens (Figure 7). These findings suggest that different *LbDof* genes may play distinct roles in the development of *L. barbarum*.

### 2.8. Expression Pattern of LbDofs Genes in L. barbarum Under Stress

**Figure 8:**
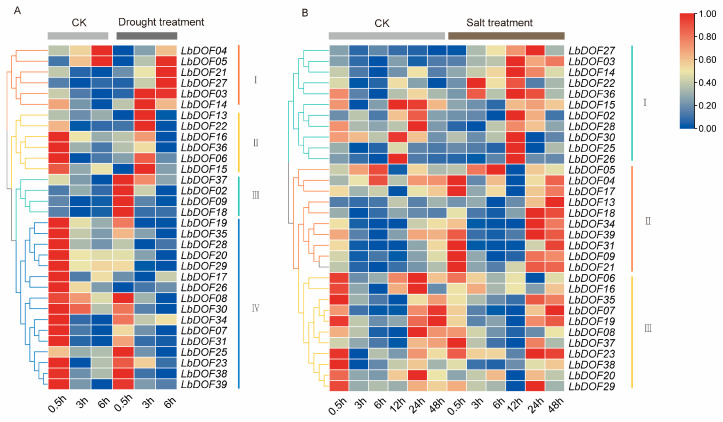
Gene expression profiles under different conditions. (**A**) Heatmap of *LbDof* gene expression in *L. barbarum* under different drought stress times (0.5 h, 3 h, 6 h); (**B**) heatmap of *LbDof* gene expression in *L. barbarum* under different times of salt stress (0.5 h, 3 h, 6 h, 12 h, 24 h, 48 h). The color scale on the right side of the heatmap indicates the relative expression level. The colors in the legend represent log2 (FPKM + 1) values in different treatment times. The red color represents the maximum value and the blue color represents the minimum value.

To understand the role of *LbDof* genes in stress responses, this study analyzed the transcriptome FPKM values of *L. barbarum* under drought and salt stress conditions. Under drought stress, the expression levels of 32 *LbDof* genes revealed that 12 *LbDofs* (*LbDof05*, *LbDof21*, *LbDof27*, *LbDof03*, *LbDof14*, *LbDof13*, *LbDof22*, *LbDof16*, *LbDof36*, *LbDof06*, and *LbDof15*) in clusters I and II showed significant up-regulation after 3 or 6 h of stress. In contrast, *LbDofs* in cluster III (*LbDof37*, *LbDof02*, *LbDof09*, and *LbDof18*) exhibited a significant increase in expression at the early stage of stress (0.6 h) (Figure 8A). Under salt stress, the expression profiles of *LbDof* genes differed from those under drought stress. Ten *LbDofs* in cluster II (*LbDof05*, *LbDof04*, *LbDof17*, *LbDof13*, *LbDof18*, *LbDof34*, *LbDof39*, *LbDof31*, *LbDof09*, and *LbDof21*) showed significant up-regulation at 24 and 48 h, while 11 *LbDofs* in cluster I (*LbDof27*, *LbDof03*, *LbDof14*, *LbDof22*, *LbDof36*, *LbDof15*, *LbDof02*, *LbDof28*, *LbDof30*, *LbDof25*, and *LbDof26*) reached their peak expression levels at 12 and 24 h. These findings suggest that *LbDof* genes may enhance salt tolerance in *L. barbarum* by positively regulating the plant’s response to salt-induced damage (Figure 8B).

### 2.9. Expression Pattern of Lbdofs During Fruit Development of Different Cultivars of L. barbarum

**Figure 9:**
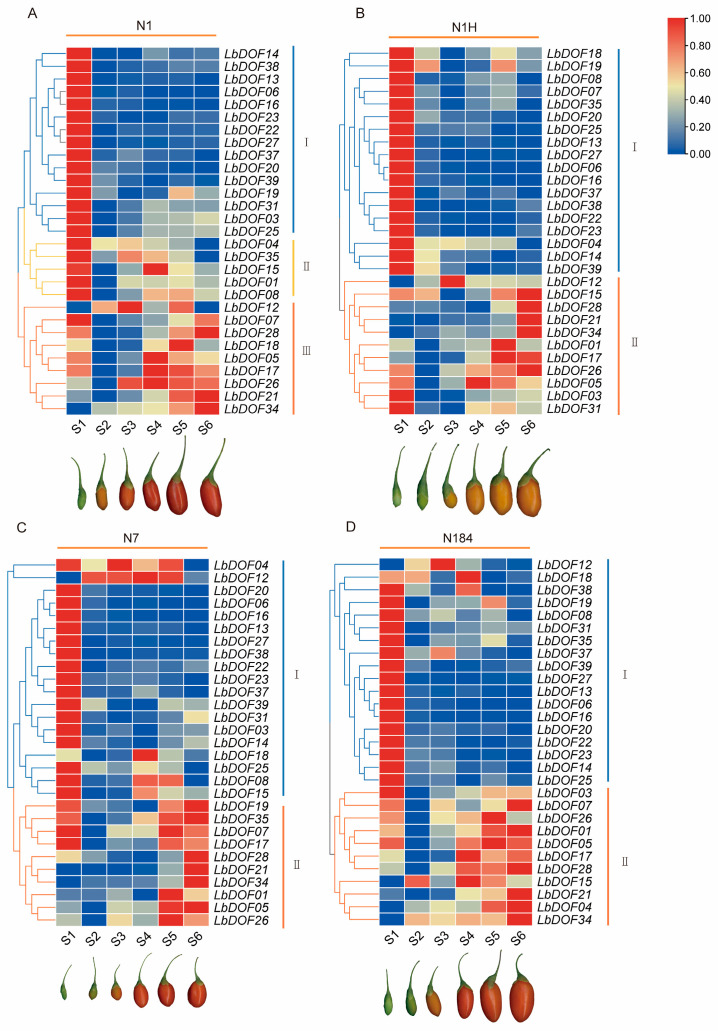
Gene expression profiles at fruit development time (S1–S6) in different varieties. (**A**) Heat map of *LbDof* gene expression in N1; (**B**) heat map of *LbDof* gene expression in N1H; (**C**) heat map of LbDof gene expression in N7; (**D**) heat map of *LbDof* gene expression in N184. The color scale on the right side of the heat map indicates the relative expression level. The colors in the legend represent log2 (FPKM + 1) values in different fruits. The red color represents the maximum value and the blue color represents the minimum value.

To investigate the role of the *LbDof* gene in the growth and development of *L. barbarum* fruit, we analyzed the gene expression profiles of four cultivars (N1, N1H, N7, and N184) across various developmental stages, from young fruit (S1) to maturity (S6). The expression patterns of *LbDof* genes were largely similar across the four cultivars. In most cases, genes in Cluster I exhibited high expression levels at the S1 stage, indicating active gene expression during the early fruit development phase. As development progressed to the S6 stage, the expression levels of many genes declined, showing a general downregulation trend, which suggests dynamic regulation of gene expression during fruit maturation. In cultivar N1, Cluster II (*LbDof04*, *LbDof35*, *LbDof15*, *LbDof01*, and *LbDof08*) showed slight upregulation in later stages (S4–S6) compared to earlier stages (S2–S3). Additionally, *LbDof12*, *LbDof07*, *LbDof18*, *LbDof17*, *LbDof26*, *LbDof21*, and *LbDof34* in Cluster II displayed a distinct upregulation pattern during S4–S6 (Figure 9A). In cultivar N1H, the expression of *LbDof12*, *LbDof15*, *LbDof28*, *LbDof21*, *LbDof34*, *LbDof01*, and *LbDof17* increased consistently throughout fruit development. Meanwhile, *LbDof26*, *LbDof05*, *LbDof03*, and *LbDof31* showed rapid accumulation at S1, declined at S2–S3, and then increased again as fruit development progressed (Figure 9B). In cultivar N7, *LbDof04* maintained high expression from S1 to S5 but dropped sharply at S6. *LbDof12* showed low expression at S1 but was highly expressed at S2–S5, followed by a decrease at S6. *LbDof19*, *LbDof35*, *LbDof07*, and *LbDof17* were highly expressed at S1, downregulated from S2 to S4, and then upregulated at S5–S6. Additionally, *LbDof28*, *LbDof21*, *LbDof34*, *LbDof01*, *LbDof05*, and *LbDof26* exhibited high expression exclusively at S5–S6 (Figure 9C). In cultivar N184, *LbDof03*, *LbDof07*, *LbDof26*, *LbDof01*, and *LbDof05* were highly expressed at S1, decreased at S2–S3, and increased again at S4–S6. Meanwhile, *LbDof17*, *LbDof28*, *LbDof15*, *LbDof21*, *LbDof04*, and LbD*of34* displayed a continuous increase in expression from S1 to S6 (Figure 9D).

## 3. Discussion

The *Dof* genes belong to a family of transcription factors unique to the plant kingdom and play a crucial role in the growth and development of nearly all plants. This study represents the first comprehensive genome-wide analysis of the *Dof* gene family in *L. barbarum*, identifying a total of 39 *LbDof* genes. The number of *Dof* genes in wolfberry is comparable to those in *A. thaliana* (47) [12], *S. lycopersicum* (33) [21], *O. sativa* (37) [15], and *P. trichocarpa* (62) [22], indicating a high degree of conservation within the *Dof* gene family across plant species, although species-specific adaptations may lead to variations in gene numbers. The encoded proteins of these *Dof* genes exhibit significant differences in length, molecular weight, and isoelectric point, suggesting their involvement in diverse physiological processes, consistent with previous studies on *Dof* genes in other species (Table 1) [15,23,24]. Analysis of exon–intron structures revealed that *LbDof* genes generally possess simple architectures, with most containing 1–3 introns and some being intronless (Figure 4B). This structural simplicity aligns with observations in other plant *Dof* gene families, implying functional optimization during evolution [6,25]. Furthermore, conserved motif analysis confirmed the presence of the characteristic C2/C2 zinc finger domain in all LbDof proteins (Figure 2), a hallmark of Dof TFs, highlighting their critical role in DNA binding and transcriptional regulation.

Phylogenetic analysis classified the LbDof proteins into 10 subfamilies (I–X) and compared them with *Dofs* from Arabidopsis, tomato, rice, and poplar (Figure 1). The results demonstrated high homology between *LbDof* and those from *Arabidopsis* and *poplar*, suggesting a shared evolutionary ancestry. This finding highlights the high conservation of *Dof* genes among dicotyledons and supports their ancient regulatory role in plant growth, development, and stress responses [26]. Notably, no tandem duplication events were detected within the *LbDof* gene family; however, 24 homologous gene pairs were identified (Figure 3C), primarily resulting from segmental duplication. This indicates that the expansion of the *LbDof* gene family likely involved unique evolutionary events, contributing to its divergence in gene number while maintaining functional conservation [27]. Segmental duplication appears to have played a pivotal role in the expansion of the *LbDof* gene family, potentially enabling the wolfberry to adapt to diverse environmental stresses and growth requirements. Such duplication events may have facilitated functional diversification, enhancing the plant’s adaptability and survival capabilities [28]. All *LbDof* gene pairs with Ka/Ks < 1 underwent purifying selection, indicating that these genes retained essential biological functions and avoided excessive mutations. This pattern aligns with the expansion mechanisms observed in other plant gene families, further supporting the significance of segmental duplication in plant genome evolution. Chromosomal distribution analysis revealed that *LbDof* genes are unevenly distributed across 11 chromosomes, with higher densities on chromosomes 1, 6, and 12, while chromosome 7 lacks any *LbDof* genes (Figure 3A). This distribution pattern, where *Dof* genes tend to cluster in specific chromosomal regions [29], may reflect functional specialization or evolutionary pressures on these regions. Motif analysis further revealed that motif 1 was uniformly present in all Dof proteins (Figure 3C), similar to observations in Arabidopsis, rice, and tomato. This suggests that LbDof TFs are evolutionarily conserved during plant development.

In other plants, *Dof* has been shown to perform various functions, including seed germination, root elongation, flowering, and fruit ripening [30]. For instance, in Arabidopsis, *Dof* regulates carbon and nitrogen metabolism [31], while in rice and tomato, they are involved in drought and salt stress regulation [19]. Given the known roles of *Dof* in other species, it is hypothesized that *LbDof* may play key roles in similar processes. Particularly in *L. barbarum*, which is renowned for its medicinal and nutritional value, these genes may have evolved to regulate fruit development and stress adaptation, aiding the plant’s survival in harsh environments.

Functional analysis of the promoter regions of *LbDof* identified an abundance of CAREs, including those associated with plant growth and development (e.g., seeds, fruits), light responses, hormone responses (e.g., ABA, GA, SA), and stress responses (e.g., drought, salt stress). The presence of these elements suggests that *LbDof* may play crucial roles in growth, development, photosynthesis, hormone signaling, and stress responses in wolfberry.

Previous studies have established a correlation between *Dof* and plant tissue differentiation as well as organ development [32]. For instance, the identification of MYB binding sites in the promoters of several *LbDof* suggests their potential involvement in regulating flavonoid biosynthesis and stress responses. However, some *LbDof* genes, such as *LbDof13*, *LbDof23*, *LbDof27*, *LbDof06*, *LbDof22*, and *LbDof16*, exhibited a gradually downregulated expression pattern during fruit development, indicating distinct roles in this process. Fruit development and ripening are complex biological processes tightly regulated by transcriptional networks involving various TFs, including NAC, MADS-box, and MYB [33]. Nevertheless, the specific roles of Dof TFs in these processes remain largely unexplored. In bananas, *MaDof10*, *MaDof23*, *MaDof24*, and *MaDof25* are ethylene-induced and show increased transcription levels during fruit ripening. MaDof23 acts as a repressor and interacts with MaERF9 to regulate ripening-associated genes [34]. Similarly, in potatoes, *StCDF1* controls tuber formation by repressing the expression of *StCO1*/*2*, thereby promoting tuber induction [35]. *Dof* also regulates plant secondary metabolism; for example, the overexpression of *FcDof4* and *FcDof16* enhances the transcription of structural genes in the flavonoid biosynthesis pathway, increasing the levels of C-glycosyl flavonoids [36]. Additionally, gene expression profiling revealed organ-specific expression patterns of *LbDof* genes across various organs, such as flowers, stems, leaves, and fruits. For example, *LbDof18* and *LbDof15* are highly expressed in flowers, while *LbDof03* and *LbDof05* are predominantly expressed in leaves, suggesting their potential roles in tissue differentiation and organ development.

In this study, we first investigated the expression profiles of all putative *LbDof* genes across various organs, including pistil, stamen, stem, immature leaves, mature leaves, immature fruits, and mature fruits. The *LbDof* genes exhibited differential expression across these organs, consistent with findings in other plant species [21,32]. Notably, *LbDof20*, *LbDof04*, *LbDof23*, *LbDof08*, *LbDof28*, *LbDof35*, *LbDof36*, *LbDof33*, *LbDof26*, *LbDof02*, *LbDof06*, *LbDof16*, *LbDof07*, *LbDof19*, LbDof22, and *LbDof27* showed relatively high expression levels in the stem, suggesting their potential involvement in stem development [31]. Furthermore, a large number of light-responsive elements, such as Box4 and G-box, were identified in the promoters of *LbDof* genes, indicating their potential roles in regulating photosynthesis and circadian rhythms [37].

Additionally, we identified *LbDof18*, *LbDof15*, *LbDof39*, *LbDof20*, *LbDof04*, *LbDof12*, *LbDof32*, *LbDof30*, and *LbDof36* as having significantly higher transcript abundance in the pistil. *AtDof4.7*, a homolog of these genes, has been implicated in the transcriptional regulation of floral organ abscission by influencing the expression of cell wall hydrolase genes [38]. Meanwhile, CDF1 functions as a repressor of *CO* gene transcription [39]. In rice, 16 *Dof* genes are expressed during grain filling and flowering, indicating their role in regulating genes essential for seed development [40]. Previous research on grapes has also demonstrated that *Dof* genes participate in different stages of fruit growth and ripening. In our study, *LbDof34* and *LbDof21* expression progressively increased from the first to the sixth stage of fruit development, peaking in mature fruit (S6), underscoring their importance in fruit maturation. Their homolog, SCAP1 (AtDof5.7), has been shown to regulate guard cell differentiation during the final stage of stomatal maturation by modulating the expression of multiple genes responsible for stomatal function [41].

The promoter regions of *LbDof* genes contain a substantial number of CAREs, including ARE, GC-motif, LTR, MBS, TCA-element, and TC-rich repeats. This suggests that these genes may play multiple roles in biotic and abiotic stress responses. Notably, *LbDof* genes exhibit significant expression changes under drought and salt stress. Specifically, *LbDof05* and *LbDof21* are markedly upregulated in response to drought stress. Under drought conditions, Dof TFs participate in various physiological and molecular responses. They regulate stomatal closure by interacting with ABA-responsive elements, thereby minimizing water loss. For example, in Arabidopsis, *AtDof4.2* is closely associated with stomatal regulation and water-use efficiency [42]. Additionally, Dof TFs contribute to root system remodeling by promoting root growth and elongation, enhancing water uptake capacity. In *O. sativa*, *OsDof3* facilitates root elongation, improving drought tolerance [43]. Dof TFs also directly regulate the expression of genes involved in the biosynthesis of osmoprotectants, such as proline and trehalose, reinforcing cellular dehydration tolerance. Overexpression of *ZmDof1* in *Z. mays* induces the accumulation of osmolytes, enhancing drought resistance [44]. Furthermore, Dof TFs coordinate with other TFs, including NAC, MYB, and WRKY, to integrate multiple signaling pathways, fine-tune drought responses, and enhance plant adaptability [45].

Conversely, *LbDof04* and *LbDof17* exhibit strong responses to salt stress, suggesting that *LbDof* may play a pivotal role in *L. barbarum* adaptation to abiotic stress. Under salt stress, Dof TFs are crucial regulators of ion homeostasis, maintaining Na^+^/K^+^ balance and preventing excessive sodium accumulation in cells [46]. For example, in rice, *OsDof15* modulates sodium transporters, improving salt tolerance [47]. Dof TFs also activate genes involved in the biosynthesis of compatible solutes, such as glycine betaine and proline, which stabilize cellular structures under salt stress. Additionally, certain Dof TFs induce morphological changes, such as inhibiting shoot growth and enhancing root elongation, aiding plant adaptation to saline environments [48]. Furthermore, Dof TFs interact with hormonal signaling pathways, including auxin, gibberellins, and ethylene, to regulate salt stress responses. Studies have shown that Dof TFs integrate with the ABA signaling pathway to fine-tune plant responses to salinity [49]. These studies highlight the multifaceted role of hormonal signaling pathways, including those mediated by Dof TFs, in regulating plant responses to salt stress. By interacting with key hormones like ethylene, auxin, and gibberellins, Dof TFs contribute to the complex network of molecular mechanisms that enable plants to survive and thrive under adverse environmental conditions.

This study focuses on the genome-wide identification and expression pattern analysis of the LbDof TFs, systematically elucidating for the first time the evolutionary characteristics, structural classification, and expression features in response to organs, stresses, and fruit development of *LbDof* in *L. barbarum*, providing key candidate genes and a theoretical framework for subsequent functional studies. However, the current research has limitations, as it has not yet validated the specific biological functions of *LbDof* members through experimental approaches such as gene knockout, overexpression, or transgenic techniques. Moreover, the expression analysis based on transcriptome data and the prediction of promoter cis-elements can only suggest potential functional associations, without directly proving the causal relationships between genes and phenotypes. Nevertheless, the evolutionary analysis, expression profiling data, and functional prediction models provided by this study significantly narrow down the scope for subsequent experimental validation, offering important prioritization criteria for the precise design of phenotypic experiments. Future research can conduct gene knockout, knockdown, and overexpression to verify gene functions, thereby comprehensively revealing the functions and regulatory mechanisms of the *LbDof* gene family.

## 4. Materials and Methods

### 4.1. Identification of LbDof in L. barbarum

The complete genome sequence, along with corresponding protein and gene annotation files for *L. barbarum*, was retrieved from the NCBI database [50]. To facilitate comparative analysis, genome sequences, protein sequences, and annotation files for *A. thaliana*, *S. lycopersicum*, *Populus trichocarpa*, and *O. sativa* were retrieved from the Ensembl Plants database [51]. From PlantTFDB version 5.0 [52], we obtained a total of 37 Dof proteins from *O. sativa*, 33 from *S. lycopersicum*, 62 from *P. trichocarpa*, and 47 from *Arabidopsis*. To identify putative members of the Dof transcription factor family in the *L. barbarum* genome, two complementary approaches were employed: a BLASTp search and a Hidden Markov Model (HMM)-based search. The HMM search utilized the PF02701 profile obtained from the EMBL-Pfam protein family database, applying default parameters. Subsequently, the presence and integrity of the conserved Dof domain in the candidate protein sequences were validated using SMART [53], EMBL-Pfam [54], and NCBI’s CD-Search [55].

### 4.2. Gene Structure and Motif Analysis

The exon–intron structures of *LbDof* genes were analyzed and visualized using TBtools software (v2.21) [56]. Conserved motifs within LbDof protein sequences were subsequently identified using the MEME Suite [57], with the maximum number of motifs specified as ten. The phylogenetic relationships, gene structural features, and conserved motif distributions were then consolidated and visualized using TBtools [56].

### 4.3. Physicochemical Characteristics and Phylogenetic Analyses

The physicochemical properties of LbDof proteins, including molecular weight (kDa), amino acid count (aa), ORF length (bp), and isoelectric point (pI), were analyzed using ExPASy [58]. Following this, the subcellular localization of LbDof proteins was predicted using WoLF PSORT [59]. To explore evolutionary relationships, the protein sequences of Dof from *L. barbarum*, *A. thaliana*, *O. sativa*, *S. lycopersicum*, and *P. trichocarpa* were aligned using MEGA 11 [60]. A Neighbor-Joining (NJ) phylogenetic tree was constructed based on the sequence alignment using 1000 bootstrap replicates, with default settings applied to all other parameters. The resulting tree was subsequently visualized using the Interactive Tree of Life (iTOL) online tool [61].

### 4.4. Cis-Regulatory Element Analysis of Lbdof Genes

A 2000 bp sequence upstream of the ATG start codon of each *LbDof* gene was extracted using TBtools software and designated as the putative promoter region. These promoter sequences were subsequently analyzed for CAREs using the PlantCARE database [56]. The distribution and types of CAREs identified were visualized using the HeatMap tool integrated within the TBtools software suite [62].

### 4.5. Collinearity Analysis and Calculation of Ka/Ks Values

MCScanX plug-in (TBtools) was utilized with default parameters to identify segmental and tandem duplication events among *LbDof* genes. Based on the chromosomal positions of these genes, TBtools was applied to conduct intragenomic collinearity analysis within the *L. barbarum* genome. Furthermore, interspecific collinearity analyses were performed between *L. barbarum* and four other plant species: *A. thaliana*, *O. sativa*, *S. lycopersicum*, and *P. trichocarpa*. The non-synonymous-to-synonymous substitution rate ratios (*Ka*/*Ks*) for collinear *LbDof* gene pairs were calculated using the *Ka*/*Ks* calculator embedded in TBtools. Divergence times were estimated using the formula T = *Ks*/2r, where Ks denotes the number of synonymous substitutions per site and r represents the rate of synonymous substitutions in plant nuclear genes. For dicotyledonous species, r was set at 1.5 × 10^−8^ substitutions per site per year [63].

### 4.6. Plant Materials and Treatments

Samples from various organs of *L. barbarum* (cultivar N1) were collected in June 2021 from three-year-old cloned seedlings cultivated in Yinchuan, Ningxia (106°9′29″ E, 38°38′51″ N, 1070 m). The collected organs included stamens, pistils, green fruits, mature fruits, stem segments, immature leaves, and mature leaves. Under standard cultivation and management conditions, three biological replicates were collected from three distinct trees to ensure experimental reliability. Immediately after collection, the samples were flash-frozen in liquid nitrogen to preserve their integrity for subsequent analysis.

For drought and salt stress treatments, seedlings were derived from laboratory-preserved *L. barbarum* (cultivar N1). Uniform stem segments were selected and inserted into rooting medium, consisting of 1 L of *Lycium*-specific medium supplemented with MS salts (4.43 g), sucrose (30 g), agar (7 g), and IBA (0.05 g). After 40 days of cultivation, morphologically uniform clonal seedlings were transferred to hydroponic culture using Hoagland solution and pre-incubated for an additional three days. All plant materials were maintained at 25 °C, with 70% relative humidity under 5000 lux light intensity, following a 16 h light/8 h dark photoperiod. Prior to treatment, the nutrient solution was completely refreshed for all seedlings and remained unchanged throughout the experiment to ensure consistency. The experiment commenced at approximately 10:00 a.m. on 24 August 2023. The control group received no treatment. For the salt treatment, Hoagland solution served as the basal medium, with 200 mM NaCl added as the sole supplement to ensure the specificity of the salt stress. Samples were flash-frozen in liquid nitrogen at 0.5 h, 3 h, 6 h, 12 h, 24 h, and 48 h post-treatment, with three biological replicates collected from three different trees to ensure statistical robustness. For drought treatment, the hydroponic solution was drained, moisture was squeezed from the sponges surrounding the root stems, and residual water was absorbed from the roots using blotting paper. Samples were flash-frozen at 0.5 h, 3 h, and 6 h post-treatment (no samples were collected at 12 h, 24 h, and 48 h due to excessive water loss), with leaf tissues collected from three individual seedlings as biological replicates.

Fruit developmental materials were collected in July 2023 from three-year-old asexual *L. barbarum* seedlings cultivated in Yinchuan, Ningxia (106°9′29″ E, 38°38′51″ N, 1070 m). The study focused on four cultivars—N1, N1H, N7, and N184—which were sampled across seven developmental stages, designated as S1 to S7. These stages included S1 (5 Days after flowering, DAF), S2 (10 DAF), S3 (20 DAF), S4 (25 DAF), S5 (30 DAF), S6 (35 DAF), and S7 (40 DAF). For each cultivar and developmental stage, fruit samples were collected from three different trees to ensure biological reproducibility, resulting in three biological replicates per stage. Immediately after collection, the samples were flash-frozen in liquid nitrogen to preserve their biological integrity. The frozen samples were then transported on dry ice to Beijing Biomarker Technologies Co., Ltd. (Beijing, China), where RNA extraction, cDNA library construction, and reference-based transcriptome analysis were conducted using the Illumina platform.

### 4.7. Expression Patterns of LbDofs

The plant materials used in this study, including stamens, pistils, green fruits, mature fruits, stem segments, immature leaves, and mature leaves of *L. barbarum*, as well as samples subjected to different stress conditions (drought and salt stress) and from various cultivars (N1, N1H, N7, and N184), were collected for transcriptome analysis. Total RNA was extracted from plant materials using the RNAprep Pure Plant Kit (Tiangen, Beijing, China), following the manufacturer’s protocol. Transcriptome sequencing and library preparation were carried out by Beijing Biomarker Technologies Co., Ltd. High-quality clean reads obtained from sequencing were aligned to the reference genome using Bowtie2 (v2.5.3) [64]. Gene expression levels were estimated using RSEM (v1.3.3) [65], with expression values expressed as fragments per kilobase of transcript per million mapped reads (FPKM). A heatmap was subsequently generated using TBtools to visualize gene expression profiles based on the calculated FPKM values.

### 4.8. Subcellular Localization

The subcellular localization experiment of *L. barbarum* was conducted using a previously established method [66], with slight modifications. Initially, specific primers for *LbDof* were designed to clone the gene. Subsequently, the CDS fragments of *LbDof04* and *LbDof38*, excluding the stop codon, were ligated into the linearized pbi121 vector and fused with the EGFP gene, yielding plasmids encoding pbi121-*LbDof04*-EGFP and pbi121-*LbDof38*-EGFP. Following vector assembly, the resulting constructs were introduced into *Agrobacterium tumefaciens* strain GV3101 using the freeze–thaw transformation method. The transformed cells were cultured overnight at 28 °C with agitation at 200 rpm. When the optical density at 600 nm (OD_600_) reached approximately 0.8, the bacterial suspension was incubated in darkness for 3 h and subsequently used for infiltration into *Nicotiana tabacum* leaves. After a 48 h incubation period under dark conditions, fluorescence signals from enhanced green fluorescent protein (EGFP) and chloroplasts were observed using a TCS SP8 X confocal laser scanning microscope (Leica, Wetzlar, Germany) with excitation at 488 nm.

## 5. Conclusions

This study provides a comprehensive genomic and functional characterization of the Dof transcription factor family in *L. barbarum*, unveiling novel insights into its dual regulatory roles in abiotic stress adaptation and developmental programming. Through systematic genome-wide screening, we identified 39 *LbDof* genes, characterized by conserved motifs, domains, and cis-regulatory elements, suggesting key regulatory functions. Phylogenetic analysis classified them into ten subfamilies, while chromosomal mapping revealed segmental duplications under purifying selection. Expression profiling demonstrated organ-specific patterns, stress-responsive upregulation, and dynamic changes during fruit maturation, supported by light- and hormone-responsive promoter elements. Nuclear localization confirmed their transcriptional roles. These findings highlight *LbDof* genes’ functional diversity, providing foundational data for the development of stress-tolerant crops and laying the groundwork for future research.

## Figures and Tables

**Table 1:** Physicochemical properties of Dof proteins from *L. barbarum*.

Gene ID	Dof Gene	Map Position (bp)	Length (AA)	PI	MW (kDa)	Num of Exon	Location
*Lba01g00421*	*LbDof01*	Chr01:9119737-9120378	213	8.14	24.31	1	Nuclear
*Lba01g00560*	*LbDof02*	Chr01:13918702-13920360	282	8.99	30.84	3	Nuclear
*Lba01g01980*	*LbDof03*	Chr01:154257739-154263430	306	5.55	33.42	2	Nuclear
*Lba01g02222*	*LbDof04*	Chr01:157935616-157936527	303	5.87	33.58	1	Nuclear
*Lba01g02268*	*LbDof05*	Chr01:158495049-158497125	449	5.72	49.27	2	Nuclear
*Lba01g02335*	*LbDof06*	Chr01:159697135-159699178	304	6.35	33.62	2	Nuclear
*Lba01g02725*	*LbDof07*	Chr01:165684285-165686213	297	7.18	32.91	2	Nuclear
*Lba01g02777*	*LbDof08*	Chr01:166398894-166400062	320	9.26	34.57	2	Nuclear
*Lba02g00233*	*LbDof09*	Chr02:4517154-4541179	659	8.53	73.62	4	Nuclear
*Lba02g00236*	*LbDof10*	Chr02:4602270-4603229	319	5.92	35.64	1	Nuclear
*Lba02g00237*	*LbDof11*	Chr02:4608282-4609223	313	5.83	35.00	1	Nuclear
*Lba02g01857*	*LbDof12*	Chr02:131223420-131224217	265	4.56	29.72	1	Nuclear
*Lba02g02528*	*LbDof13*	Chr02:147141275-147141931	218	6.12	22.66	1	Nuclear
*Lba03g00541*	*LbDof14*	Chr03:10678916-10680566	393	9.3	41.85	3	Nuclear
*Lba03g01659*	*LbDof15*	Chr03:121309151-121310338	261	8.76	28.50	2	Nuclear
*Lba03g02183*	*LbDof16*	Chr03:133102286-133104176	300	6.66	33.36	2	Nuclear
*Lba03g02450*	*LbDof17*	Chr03:138661121-138664401	500	6.14	54.38	2	Nuclear
*Lba03g02993*	*LbDof18*	Chr03:146745940-146746503	187	9.34	20.29	1	Nuclear
*Lba04g00060*	*LbDof19*	Chr04:794018-796171	323	8.82	35.30	3	Nuclear
*Lba05g00239*	*LbDof20*	Chr05:4035257-4036339	307	9.37	34.08	2	Nuclear
*Lba05g02493*	*LbDof21*	Chr05:142942613-142944872	401	5.96	44.32	2	Nuclear
*Lba06g00549*	*LbDof22*	Chr06:12304979-12306315	277	6.38	31.26	2	Nuclear
*Lba06g00550*	*LbDof23*	Chr06:12308649-12310464	281	8.08	31.61	2	Nuclear
*Lba06g00551*	*LbDof24*	Chr06:12350155-12352434	252	8.83	28.82	2	Nuclear
*Lba06g00700*	*LbDof25*	Chr06:18846366-18847463	298	9.43	32.64	2	Nuclear
*Lba06g01885*	*LbDof26*	Chr06:109723965-109725506	287	9.19	30.91	3	Nuclear
*Lba08g00407*	*LbDof27*	Chr08:12244232-12246259	310	6.19	34.88	2	Nuclear
*Lba08g01387*	*LbDof28*	Chr08:117156146-117157413	356	9.3	38.46	2	Nuclear
*Lba09g02477*	*LbDof29*	Chr09:125873081-125874140	252	7.55	28.00	2	Nuclear
*Lba10g00246*	*LbDof30*	Chr10:4459414-4461261	356	8.33	38.40	3	Nuclear
*Lba10g01565*	*LbDof31*	Chr10:97856988-97859756	424	6.72	46.50	2	Nuclear
*Lba11g02467*	*LbDof32*	Chr11:119952692-119957326	303	9.13	34.16	3	Nuclear
*Lba12g00530*	*LbDof33*	Chr12:63267215-63267769	184	9.73	19.88	1	Nuclear
*Lba12g00627*	*LbDof34*	Chr12:66014533-66017295	452	5.24	49.63	2	Nuclear
*Lba12g01572*	*LbDof35*	Chr12:84113552-84114904	395	7.76	43.91	2	Nuclear
*Lba12g01573*	*LbDof36*	Chr12:84129012-84129893	293	9.81	31.83	1	Nuclear
*Lba12g01646*	*LbDof37*	Chr12:85470852-85471736	294	5.53	33.26	1	Nuclear
*Lba12g02508*	*LbDof38*	Chr12:99924661-99925524	287	9.14	31.10	1	Nuclear
*Lba12g02669*	*LbDof39*	Chr12:102326211-102328664	446	6.03	49.84	2	Nuclear

## Data Availability

The datasets supporting the results presented in this manuscript are included within the article (and its Supplementary Materials). The RNA seq data reported in this paper have been deposited in the China National Center for Bioinformation/Beijing Institute of Genomics, Chinese Academy of Sciences (PRJCA025572, https://ngdc.cncb.ac.cn/bioproject/browse/PRJCA025572, accessed on 25 April 2024).

## Supplementary Materials

The following supporting information can be downloaded at https://www.mdpi.com/article/10.3390/plants14111567/s1, Supplementary Table: Table S1. The protein sequences of all the Dof genes from *L. barbarum*, *O. sativa*, *S. lycopersicum*, *P. trichocarpa*, and *A. thaliana* used in this study; Table S2. The multicollinearity and related information of *LbDof* genes with the genomes of *A. thaliana*, *O. sativa*, *P. trichocarpa* and *S. lycopersicum*; Table S3. *Ka*/*Ks* analysis and estimated divergence time for tandem and segmental duplicated *LbDof* genes; Table S4. The *LbDof cis*-regulatory elements in 2000 bp upstream promoter regions.

## Abbreviations

BLASTp: Basic Local Alignment Search Tool for proteins; HMM: Hidden Markov Model; CAREs: *Cis*-acting regulatory elements; TF: transcription factor; Dof: DNA-binding with One Finger; *CO*: CONSTANS; CDF: cycling Dof factor; DAF: Days After Flowering; NJ: neighbor-joining; FPKM: fragments per kilobase of transcript per million; MW: molecular weight.
